# Immuno-informatics profiling of monkeypox virus cell surface binding protein for designing a next generation multi-valent peptide-based vaccine

**DOI:** 10.3389/fimmu.2022.1035924

**Published:** 2022-11-02

**Authors:** Maha Yousaf, Saba Ismail, Asad Ullah, Shabana Bibi

**Affiliations:** ^1^ Department of Biosciences, COMSATS University Islamabad, Islamabad, Pakistan; ^2^ Department of Biological Sciences, National University of Medical Sciences, Rawalpindi, Pakistan; ^3^ Department of Health and Biological Sciences, Abasyn University, Peshawar, Pakistan; ^4^ Department of Biosciences, Shifa Tameer-e-Millat University, Islamabad, Pakistan; ^5^ Yunnan Herbal Laboratory, College of Ecology and Environmental Sciences, Yunnan University, Kunming, China

**Keywords:** monkeypox virus, molecular docking, next generation multi-valent vaccine, peptide vaccine, molecular dynamic simulation assay

## Abstract

Monkeypox is a viral etiological agent with hallmarks analogous to those observed in smallpox cases in the past. The ongoing outbreak of Monkeypox viral infection is becoming a global health problem. Multi-valent peptide based next generation vaccines provides us a promising solution to combat these emerging infectious diseases by eliciting cell-mediated and humoral immune response. Considering the success rate of subtractive proteomics pipeline and reverse vaccinology approach, in this study, we have developed a novel, next-generation, multi-valent, *in silico* peptide based vaccine construct by employing cell surface binding protein. After analyzing physiochemical and biological properties of the selected target, the protein was subjected to B cell derived T cell epitope mapping. Iterative scrutinization lead to the identification of two highly antigenic, virulent, non-allergic, non-toxic, water soluble, and Interferon-gamma inducer epitopes i.e. HYITENYRN and TTSPVRENY. We estimated that the shortlisted epitopes for vaccine construction, roughly correspond to 99.74% of the world’s population. UK, Finland and Sweden had the highest overall population coverage at 100% which is followed by Austria (99.99%), Germany (99.99%), France (99.98%), Poland (99.96), Croatia (99.93), Czech Republic (99.87%), Belgium (99.87), Italy (99.86%), China (97.83%), India (97.35%) and Pakistan (97.13%). The designed vaccine construct comprises of 150 amino acids with a molecular weight of 16.97242 kDa. Molecular docking studies of the modelled MEMPV (Multi-epitope Monkeypox Vaccine) with MHC I (PDB ID: 1I1Y), MHC II (PDB ID: 1KG0), and other immune mediators i.e. toll like receptors TLR3 (PDB ID: 2A0Z), and TLR4 (PDB ID: 4G8A) revealed strong binding affinity with immune receptors. Host immune simulation results predicted that the designed vaccine has strong potency to induce immune responses against target pathogen in the form of cellular and antibody-dependent immunity. Our findings suggest that the hypothesized vaccine candidate can be utilized as a potential therapeutic against Monkeypox however experimental study is required to validate the results and safe immunogenicity.

## Introduction

Monkeypox is a viral zoonotic disease with characteristics similar to those observed in smallpox cases in the past, however, it is clinically less virulent than smallpox (1). It is caused by monkeypox virus, a member of the family Poxviridae’s Orthopoxvirus genus (2–4). The clinical syndrome is characterized by fever, rash, headache, flu and lymphadenopathy (5). Complications of monkeypox can include pneumonitis, encephalitis, sight-threatening keratitis, and secondary bacterial infections (6). Respiratory excretions, contact with outside fabric or exposure to lesion exudate are considered as mode of transmissions for monkeypox infection (7, 8).

In 1970, first case of monkeypox disease was reported in congo (9, 10). Seven endemic nations reported 1408 suspected and 44 confirmed cases, resulting in 66 fatalities, between January and June 1, 2022 (11). Monkeypox is persistent in a number of countries, including Cameroon, the Central African Republic, the Democratic Republic of the Congo, Gabon, and Ghana (identified in animals only) (11). According to WHO, the situation is evolving and more cases of monkeypox will be found as the epidemic spreads and surveillance improves in both endemic and non-endemic countries (11).

Uptil now, no proper medication has been developed and commercialized to tackle with human monkeypox disease. In past decades, Dryvax, a small pox vaccine was utilized against both small pox and monkeypox (12), however negative side effects shown by vaccinated individuals banned its usage (13, 14). In 2019, Jynneos was scientifically and experimentally approved by Food and Drug Administration US for monkeypox and smallpox prevention (15). Although Jynneos is a safe vaccine and can be utilized under emergency situations, but it is important to remember that since it is not derived from the monkey pox virus itself, it may lose its efficacy if the virus undergoes radical changes (16). According to recent research, Tecovirimat and Brincidofovir mainly responsible as for the treatment human smallpox disease, can play the role of a promising therapeutic against monkeypox disease temporarily. Integration of immune-informatics in vaccine development offers a rapid, accurate, and efficient method for creating disease vaccines (17). Pathogen secretory proteins are a great candidate for predicting B and T cell epitopes in the development of vaccines due to their antigenicity (18, 19). The aim of this research is to construct a novel multi-epitope vaccine responsible to elicit humoral and cell mediated immune response against human monkeypox by extracting highly immunogenic epitopes from cell surface binding protein using an *in silico* immune-informatics pipeline. Cell surface binding protein enables virion adhesion to the target cell by binding to chondroitin sulfate on the cell surface and resides in the outer-membrane of the microbe, making it a potential candidate for vaccine designing.

## Workflow

**Figure 1:**
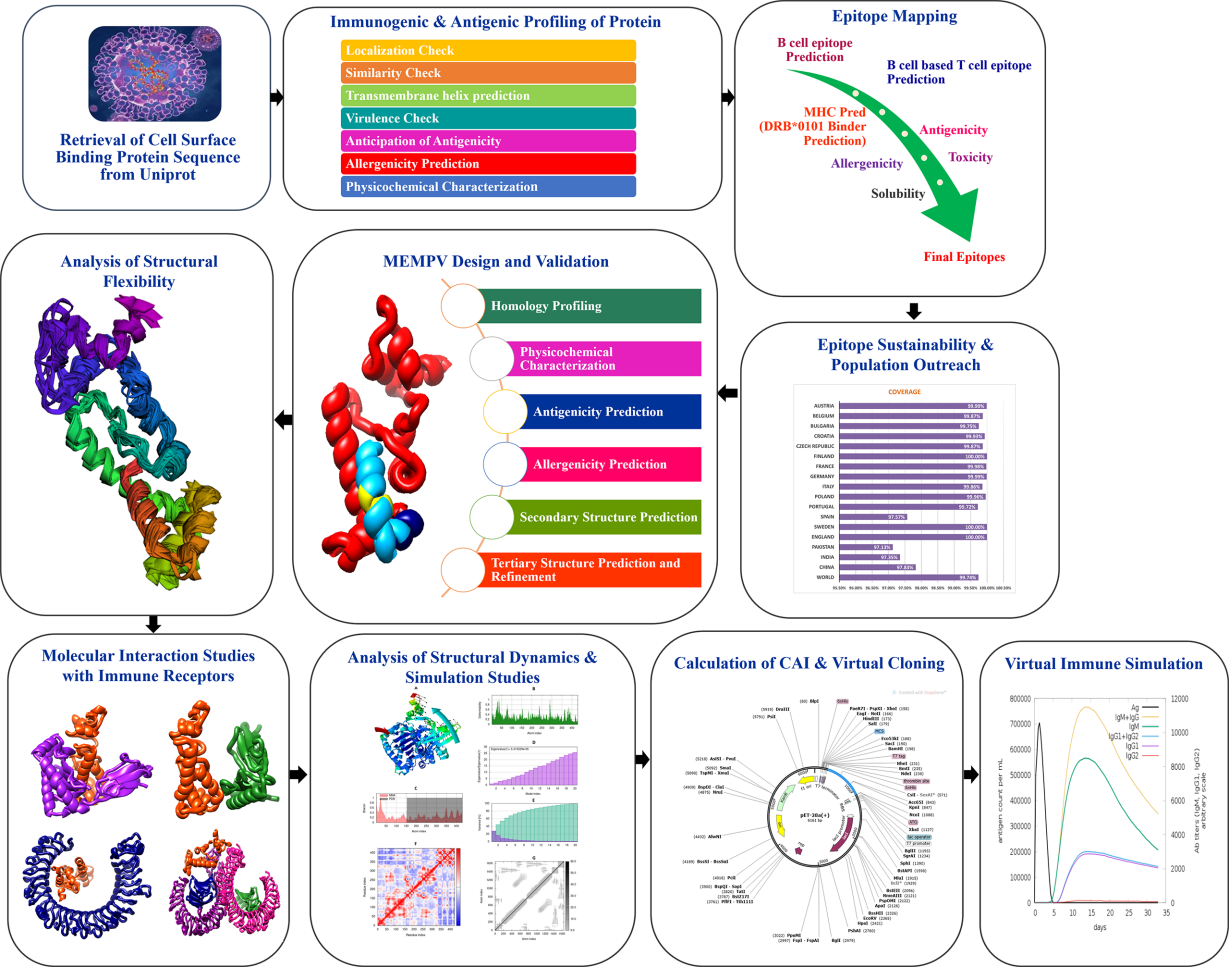
Diagram showing the framework that was created to highlight prospective monkeypox vaccine candidate, followed by the anticipation of antigenic B cell derived T cell epitopes. A chimeric vaccine made up of overlapping pooled epitopes and adjuvant was created during the MEMPV’s design and post processing steps. This designed construct was 3D modelled, structurally refined and optimized for codon usage, and cloned in an expression vector. The molecular docking and refining phases were especially focused on determining the vaccine construct’s preferred binding mode with the immunological receptors MHC I, MHC II, TLR3 and TLR4 that allowed favourable contacts for stable complex formation. Moving further, vaccine-immune receptor complex behavior was depicted by performing a normal mode analysis of the top hit docked complexes.

**Figure 1** shows the whole process of the substantial *in silico* research carried out in this study to develop a Multi-epitope Monkeypox Vaccine (MEMPV).

### Protein profiling and epitope mapping

The primary sequence of Cell Surface Binding Protein (Uniprot accession number: Q3I8Q9) of monkeypox virus was obtained from UniProt for epitope screening. The immunogenic and antigenic profile of the chosen protein was assessed. In order to determine its likelihood of allergenicity, antigenicity, and physicochemical characteristics, Allertop 2.0 (20), VaxiJen (21) and Protparam (22) were employed respectively. TMHMM2.0 web-tool was employed to calculate number of transmembrane helices in the targeted protein (23). Anticipation of virulence was performed by subjecting the target to VirulentPred web-server (24). In order to avoid auto-immune reactions, targeted protein was passed through similarity check with Human and proteome proteome *via* BLASTp (https://blast.ncbi.nlm.nih.gov/Blast.cgi?PAGE=Proteins). Both B cell and T cell epitope predictions were made using the Immune Epitope Data Base (IEDB) server (25). Bepipred Linear Epitope Prediction 2.0 algorithm (26) was employed to anticipate linear B cell epitopes, and those with a prediction score>0.5 were taken into account for mapping T cell epitopes. According to their significant correlation with a reference set of MHC I and MHC II alleles, T cell epitopes were selected for further processing. High-affinity MHC allele binders are epitopes with minimal percentile scores (cut off ≤ 40). Filtered epitopes were then subjected to MHCPred 2.0 (27) for the evaluation of their probable binding affinity to the widely distributed HLA II DRB*0101 allele in different populations. Those with an IC50 value<100 nM were classified as effective binders of DRB*0101 (28). With a threshold value of 0.5, antigenic epitopes were predicted by VaxiJen 2.0 (21). ToxinPred (29) was used to eliminate toxic peptides, however, AllerTop 2.0 (30) was utilized to reveal allergic epitopes. The filtered non-toxic epitopes were analyzed by an IFN epitope server to determine their propensity to elicit IFN-gamma responses (31), and only IFN-gamma positive epitopes were selected for further investigation.

### Epitope sustainability and estimation of population coverage

The IEDB population coverage analysis tool was employed to study the coverage of the anticipated epitopes in the global human population as it is since we believed the vaccine we developed to be efficacious for a significant portion of the human population (25).

### Chimeric MEMPV construction

In comparison to conventional vaccines or single-epitope vaccines, multi-epitope vaccine constructs (MEPVC) are robust (32, 33). They are thought to simultaneously elicit strong and broad-spectrum humoral and cellular immune responses (34). These vaccines are frequently conjugated with adjuvants, which is thought to create prolonged immune responses and boost immunogenicity while minimizing the presence of any unwanted component that can cause pathological immune reactions or negative side effects. In this study, through the use of AAY linker, filtered B cell based T cell epitopes, Cholera Toxin B adjuvant and EAAAK linker, a state of the art, next generation multi-epitope vaccine (MEMPV) is developed.

### Physiochemical evaluation, immunological assessment and Modeling of MEMPV’s chimera

The ProtParam tool (22) of the ExPASy server predicted the physicochemical characteristics. SCRATCH predictor server’s 3Dpro programme was employed to model the three-dimensional (3D) structure of the MEMPV from scratch as there is no template available (35). Following that, using GalaxyLoop from GalaxyWeb (36), loop modelling was carried out in the 3D structure of MEMPV. Refinement of the predicted 3D structure is essential to remove bad contacts, therefore GalaxyRefine was employed to execute this purpose (37).

### Assessment of structural flexibility

Flexible structural design is essential for MEMPV’s optimum functioning and molecular recognition. Utilizing CABS-Flex 2.0 web server (38), we performed a coarse-grained simulation of MEMPV to analyze its structural flexibility. On the CABS-Flex 2.0 web server, parameters for the MEMPV flexibility studies included 50 number of cycles, 8954 RNG seed, 50 cycles between trajectories, global C-alpha restraints weight (1.0), and global side-chain restraint weight (1.0).

### MEMPV-MHC I, MHC II, TLR3, TLR4 binding interaction studies: A blind docking protocol

Blind molecular docking studies were performed to study MEMPV’s affinity for immune receptors as an agonist (39). To execute this purpose, an online PatchDock server was employed (40), where MHC I (PDB ID: 1L1Y), MHC II (PDB ID: 1KG0), TLR3 (PDB ID: 1ZIW) and TLR4 (PDB ID: 4G8A) were chosen as immune cell receptors because of their potential to elicit immune response against foreign pathogens and MEMPV construct was selected as ligand. The default clustering RMSD value is 4.0. Fast Interaction Refinement in Molecular Docking (FireDock) server was utilized to improve the interactions in the output docked solutions (41, 42). UCSF Chimera 1.13.1 was used to thoroughly visualize the MEMPV’s conformation of the chosen complex with regard to MHC I, MHC II, TLR3 and TLR4 (43).

### Stability, flexibility and dynamics of the MEMPV-immune receptors:

In order to analyze complex’s stability, three dimensional flexibility and structural dynamics of MEMPV-immune receptor docked cluster, normal mode analysis was performed. In particular, the methods of molecular dynamics (MD) and normal mode analysis (NMA) are useful for defining many dynamic aspects of biological macromolecules. Even for large proteins and protein complexes with experimentally or *in silico* determined structures, NMA may be used to swiftly and systematically study protein flexibility and dynamics since it is computationally less expensive than MD. NMA is particularly useful for describing the flexible states that proteins assume around an equilibrium location. These conditions have consistently been demonstrated to have functional importance and biological relevance. By contrasting the dynamic behavior of the protein(s) with normal modes, the stability of the protein(s) can be evaluated (44, 45). It is a significant technique that might be used instead of all-atom molecular simulation, which is computationally demanding (46, 47). The total motion of proteins was investigated by carefully examining the normal modes associated with internal coordinates (48). When compared to widely used molecular dynamic (MD) simulations, the method adopted is recognized to produce effective results in less time (49, 50). Thus, utilizing the iMOD server, the movements of protein complexes were examined while important aspects including eigenvalues, b factors, covariance, and deformability were taken into consideration. The deformability at the level of the residue determines how easily protein chains can be bent. Each normal mode’s eigenvalue provides details about the stiffness of the motion. Additionally, this offers vital information about the energy required to distort the protein(s) structure (s). Low eigenvalues (51) are a blatant indicator of the simpler deformation.

### Calculation of codon adaptation index and virtual cloning

The MEMPV sequence was reversibly translated in order to achieve a high production rate utilizing the Escherichia coli K12 expression system (52). The JCat web-tool (53) was employed for this, and the codon adaptation index (CAI) was utilized to assess the cloned MEMPV expression rate. The vaccine’s DNA sequence was then computationally cloned into the pET28a (+) expression vector *via* SnapGene software.

### Virtual immune simulation of MEMPV

To computationally analyze the MEMPV’s ability to activate the host immune system, C-ImmSim server (https://kraken.iac.rm.cnr.it/C-IMMSIM/) (54) was used. The host immunological responses against an antigen are assessed using this server’s machine learning and position-specific scoring matrix (PSSM) (54). The lymph nodes, bone marrow, and thymus are the three anatomical components that are connected to the human immune system. The following values were used as input parameters for the immunological simulations: 100 steps, 10 volumes, 12345 random seeds, HLA (A0101, A0101, B0702, B0702, DRB1 0101, DRB1 0101), 3 injections, and default settings for all other features.

## Results

### Immunogenic and physicochemical profiling of cell surface binding protein

To develop a potential MEMPV, it is mandatory for the targeted protein to reside in outer membrane or periplasmic membrane of the cell. Along with sub-cellular localization, presence of no more than one transmembrane helices, no sequence homology with human and mouse, non-allergenicity, high antigenicity (cut-off >0.4), molecular weight<110kDa and virulence factor>0.5 make the protein a promising candidate for vaccine construct development. Physicochemical evaluation of cell surface binding protein was performed by ExPASy protparam web-tool (22) which predicted the protein to be stable. AllerTop 2.0 (30) anticipated non-allergenic behavior of said protein target. Result of VaxiJen web-server (21) showed the protein to be highly antigenic (cut-off score>0.4). To predict the virulence of protein virulentpred web tool was employed (24). **Table 1** provides a detail description of immunogenic and physicochemical profiling of cell surface binding protein.

**Table 1:** Immunogenic and physicochemical profile of cell surface binding protein.

Property	Value
Sub-cellular localization	Virion Membrane protein
Transmembrane helices count	1
Allergenicity	Non-allergen
Antigenicity	0.5311
Virulence based on Amino Acid Sequence	Virulent (1.3778)
Number of Amino Acids	304
Molecular Weight	35278.02 Daltons
Theoretical pI	7.77
Negatively Charged Residues	31
Positively Charged Residues	32
Chemical Formula	C1614H2454N412O468S5
Half life in mammals	30 hours
Half life in yeast	20 hours
Half life in mammals	30 hours
Gravy	-0.367
Aliphatic Index	87.89

### Epitope mapping

Acquired immune responses, which are systemic and highly specialized, aid the immune system eliminate or stop the spread of infections (55). Prioritization of the potential epitopes started by prediction of B cell epitopes by IEDB (25). 13 B cell epitopes were anticipated from the target protein consequently leading to the derivation of 18 T cell epitopes **(** **Supplimentary Table 1** **).** These B cell based T cell epitopes were then scrutinized and shortlisted on the basis of their binding affinity with the most prevalent allele among *Homo Sapiens* i.e. DRB1*0101 (IC50 cut-off value <100), antigenicity, allergenicity, toxicity, water solubility and IFN-gamma production. Epitopes having the capacity to interact with and bind to DRB1*0101 allele induce robust immunological responses (56). By choosing the epitopes with the lowest IC50 value, particularly those with a value under 100 nM, the prediction accuracy was guaranteed (28). All epitopes with an IC50 of 100 nM for T cell alleles are considered as a high binding molecules based on the competitive binding test (57). Antigenic profiling of DRB1*0101 allele binding epitopes was re-performed in order to ensure their capacity to bind immune cell receptor (58). Allergic sequences were eliminated from the list to prevent vaccine-related allergies. Additionally, the potential for virulence, water solubility, and toxicity were all re-assessed. Following the analysis outlined above, a total of two B cell-based T cell epitopes i.e. HYITENYRN and TTSPVRENY were chosen for MEMPV’s development. **Supplimentary Table 2** illustrates about the screened B cell derived T cell epitopes.

### Assessment of population outreach

**Figure 2:**
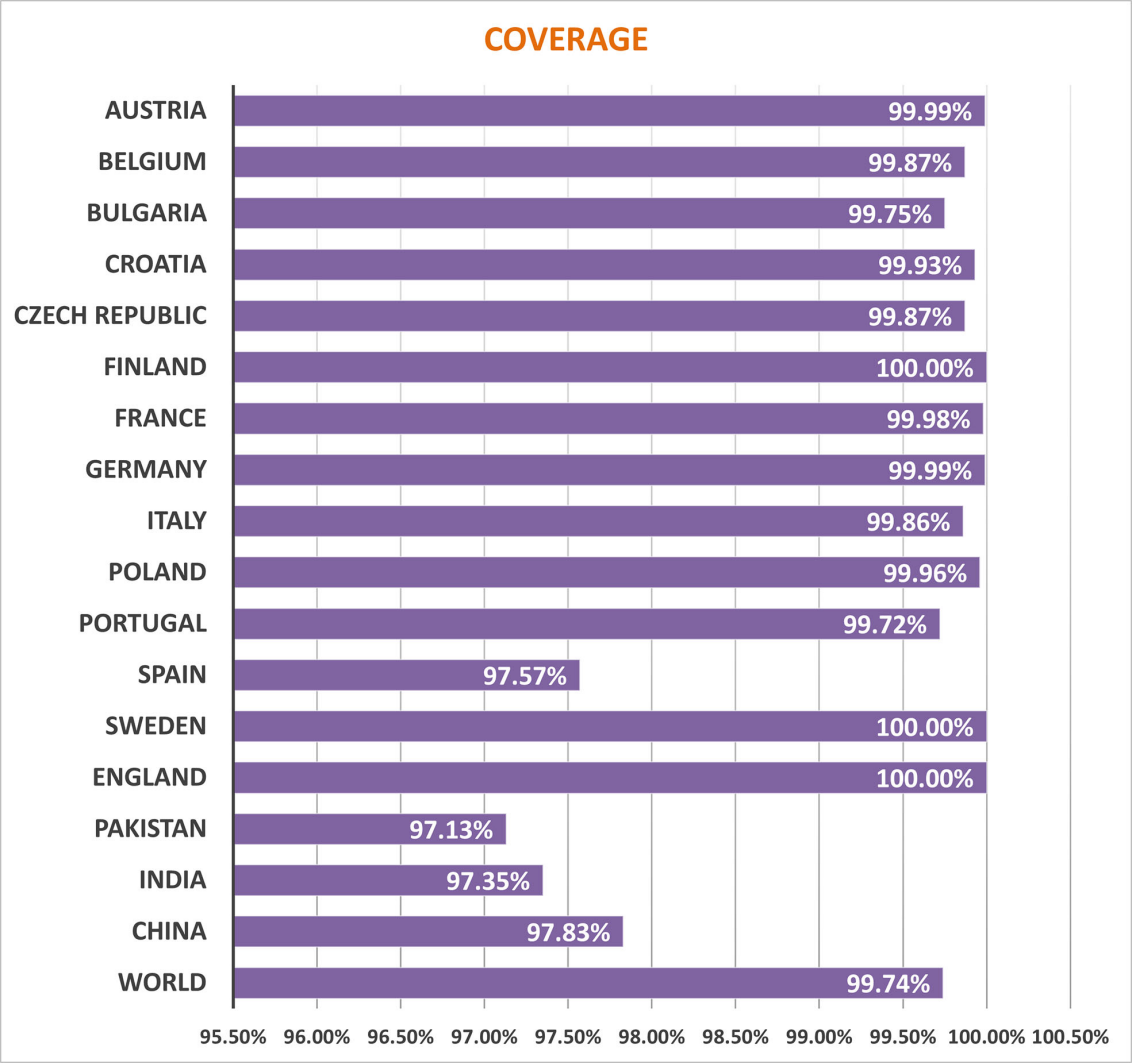
Population outreach of screened epitopes.

Several HLA alleles and their expressions have demonstrated striking worldwide dispersion at multiple frequencies in various ethnic groups and nations. Therefore, the distribution of HLA alleles is essential for the development of an effective MEMPV construct. We estimated that the prioritized epitopes roughly correspond to 99.74 percent of the world’s population. UK, Finland and Sweden had the highest overall population coverage at 100%, followed by Austria (99.99%), Germany (99.99%), France (99.98%), Poland (99.96), Croatia (99.93), Czech Republic (99.87%), Belgium (99.87), Italy (99.86%), China (97.83%), India (97.35%) and Pakistan (97.13%) **(** **Figure 2** **)**. In summary, our research proved that the selected epitopes would be strong candidates for creating a MEMPV construct.

### MEMPV construct design and post-processing

MEPVCs are stable, specific, cost-effective, time-saving, and stable molecules with the added perk of not having the entire pathogen (59). They have the capacity to concurrently elicit massive and broad-spectrum humoral and cell mediated immune response as they include a substantial number of T cell and B cell epitopes. The two nominated epitopes were linked together *via* flexible AAY linkers. AAY has the potential to boost the immunogenicity of the peptide-based vaccination, according to recent experimental research (60, 61). Cholera toxin b (CTB) was employed as an adjuvant and joined with epitopes at N-terminus *via* EAAAK linker to enhance the antigenicity of the designed construct. EAAAK is a rigid linker responsible to provide firmness to the vaccine’s molecular structure. CTB is a harmless component of cholera toxin, that binds to common antigen-presenting cells such dendritic cells, B cells, and macrophages (62). One of our MEMPV construct’s most desirable characteristics is that it will provide the highest accessibility and contact with the immune system within the human body.

### Physicochemical profiling and analysis of immunogenicity

According to assessment of its physical and chemical properties, the newly established vaccine construct has 150 amino acids and a molecular weight of 16.97242 kDa. Scientifically, vaccines with a molecular weight<110 kDa are regarded as robust and effective (28). Theoretical isoelectric point is found to be 8.8, which falls within the normal pH range. In human reticulocytes (*in vitro*), yeast (*in vivo*), and Escherichia coli, the MEMPV’s sequence’s half-life was determined to be 30 hours, more than 20 hours, and more than 10 hours, respectively (*in vivo*). The instability index was calculated to be 37.01 indicating stability of our designed construct. Our vaccine’s aliphatic index, which was estimated to be 82.73, is a significant indicator of its thermo-stability at a range of temperatures. According to physicochemical analysis, the GRAVY score for hydrophobicity is -0.263 indicating that the designed construct is hydrophilic (desired attribute) in nature and has the capacity to interact favorably with water molecules making it a suitable vaccine. Antigenicity of the construct is re-evaluated and computed to be 0.5199. AllerTop 2.0 confirmed the construct to be non-allergenic. Immunological profile of vaccine construct along with its physicochemical characteristics is described in **Table 2**.

**Table 2:** Immunogenic and physicochemical profile of proposed MEMPV.

Characteristic	Value
No. of Amino acids	150
Molecular Weight	16.97242kDa
Theoretical iso-electric point	8.8
Instability Index	37.01
Stability	Stable
Aliphatic Index	82.73
GRAVY score for hydrophobicity	-0.263
Half Life in human reticulocytes	30 hours
Half Life in yeast (*in vivo*)	More than 20 hours
Half Life in Escherichia coli	More than 10 hours
Antigenicity	0.5199
Allergenicity	Non-allergen

### Anticipation of secondary structure, 3D modeling and refinement:

The 3D Scratch pro (35) was used to obtain the multi-epitope vaccine’s first 3D structure. Limited by the lack of high-quality PDB templates to utilize as a guide for the structure prediction process, this programme uses a *de novo* approach (structural templates are not employed). After that, the model was sent to the GalaxyRefine server (37) for both refinement of local regions and global structural enhancement **(** **Table 3** **)**. Based on galaxy refinment scores, model 1 with GDT-HA of 0.95, RMSD of 0.429, MolProbity of 2.178, clash score of 25.5, 1.6% poor rotamers and 97.3% rama-favoured regions was chosen for secondary structure analysis *via* SOPMA (63).

**Table 3:** Description of Galaxy Refine’s improved structural models.

Model	GDT-HA	RMSD	MolProbity	Clash score	Poor Rotamers	Rama-favored
Initial	1	0	3.424	98.6	3.1	88.5
MODEL 1	0.95	0.429	2.178	25.5	1.6	97.3
MODEL 2	0.9267	0.464	2.07	23	0.8	96.6
MODEL 3	0.9467	0.425	1.972	20.9	1.6	98
MODEL 4	0.9317	0.482	2.254	25.1	1.6	96.6
MODEL 5	0.9383	0.474	2.097	20.5	2.4	98

**Figure 3:**
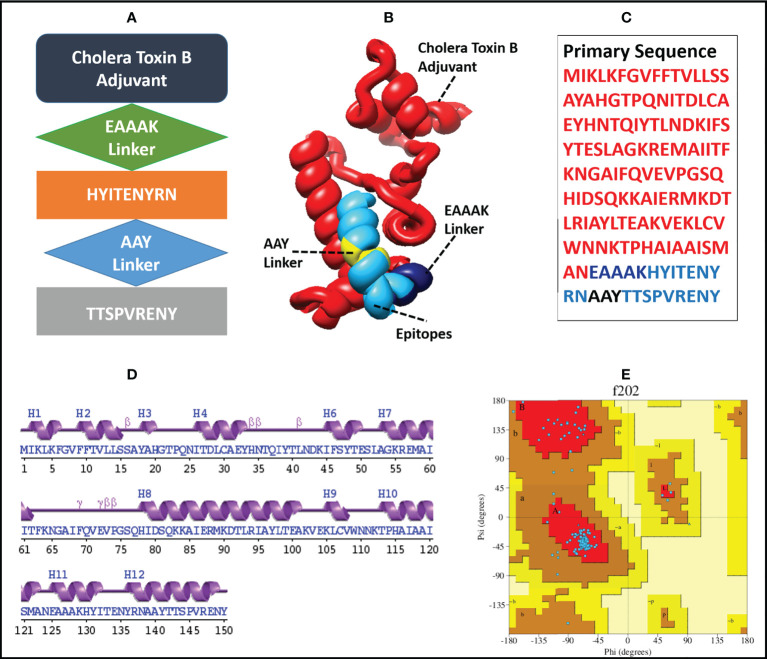
Diagrammatic illustration of the finalized MEMPV structural model; **(A)** a graphical representation of the arrangement of the selected epitopes, linkers, and adjuvant **(B)** Refined three-dimensional structure of the MEMPV (Cholera Toxin B adjuvant in red color, EAAAK linker in navy blue, AAY linkers in yellow, and epitopes in deep sky blue color); **(C)** Primary sequence of vaccine construct; **(D)** Secondary structure characterization;**(E)** Ramachandran plot illustration of improved MEMPV design.

The finalized MEMPV construct contains 50.67% alpha helices, 18% beta-strands, 26% random coils and 5.33% beta turns. However, no 3_10_ helix, Pi helix, beta-bridge and bend region was observed in its secondary structure. According to Ramachandran plot analysis carried out by using PROCHECK (64), the refined model has 127 (91.4%) residues in the most favoured regions and 12 (8.6%) residues in additionally allowed regions. However, no residue was observed in generously allowed and disallowed regions. A comprehensive description of the enhanced MEMPV assembly is shown in **Figure 3**.

### Assessment of structural flexibility

**Figure 4:**
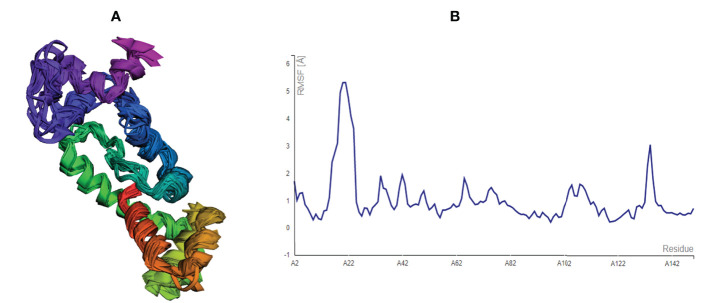
Structural flexibility assessment of the improved MEMPV: **(A)** 10 superposed MEMPV models **(B)** Root mean square fluctuation (RMSF) findings of the proposed MEMPV construct.

After modelling and refinement, the proposed MEMPV underwent structural flexibility analysis using the CABS-flex 2.0 server, which produced 10 alternative models (38). The range of the root mean square fluctuation (RMSF) was 0.2080 (lowest) to 5.3130 (highest) (**Figure 4**). These outcomes demonstrated that the vaccine construct we created is suitable for further processing.

### Analysis of binding interactions (MEMPV-immune receptors)

**Figure 5:**
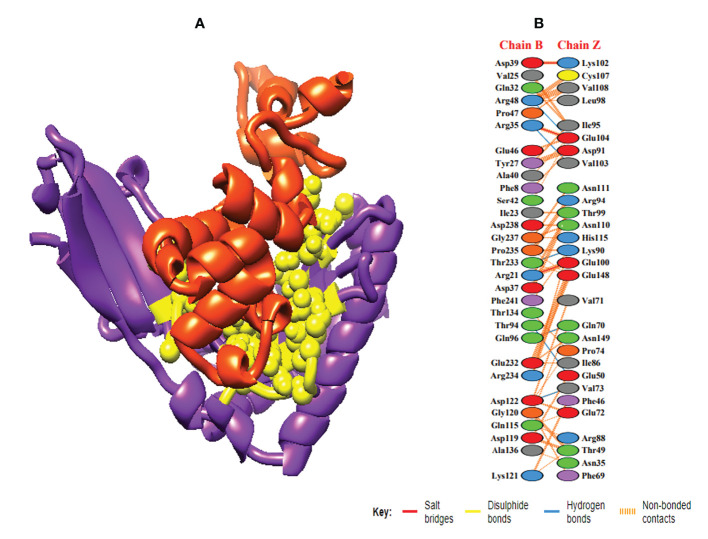
Schematic illustration of MEMPV-MHC I docked cluster. **(A)** Docked pose of MEMPV-MHC I docked cluster. Construct in orange-red, MHC I receptor in purple and interacting residues between immune receptor and vaccine are shown in yellow color. **(B)** Dimplot of vaccine construct-MHC II docked complex.

**Figure 6:**
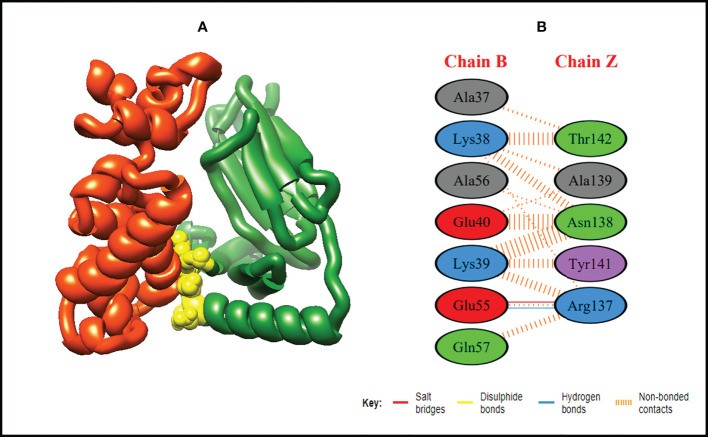
Schematic illustration of MEMPV-MHC II docked cluster. **(A)** Docked pose of MEMPV-MHC II docked cluster. Construct in orange-red, MHC II receptor in forest green and interacting residues between immune receptor and vaccine are shown in yellow color. **(B)** Dimplot of vaccine construct-MHC II docked complex.

**Figure 7:**
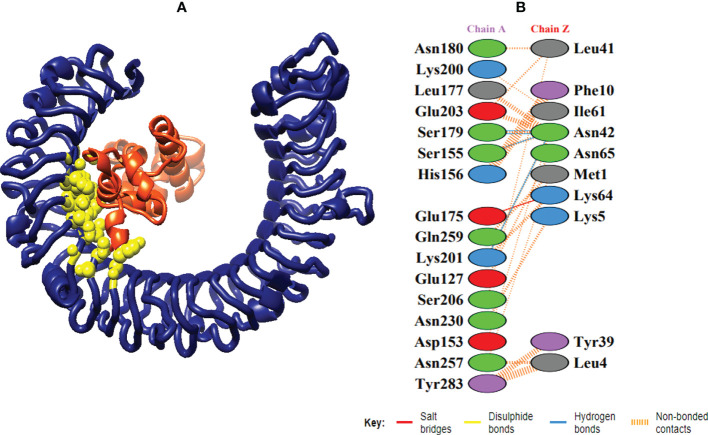
Schematic illustration of MEMPV-TLR3 docked cluster. **(A)** Docked pose of MEMPV-TLR3 docked cluster. Construct in orange-red, TLR3 receptor in navy blue and interacting residues between immune receptor and vaccine are shown in yellow color. **(B)** Dimplot of vaccine construct-TLR3 docked complex.

**Figure 8:**
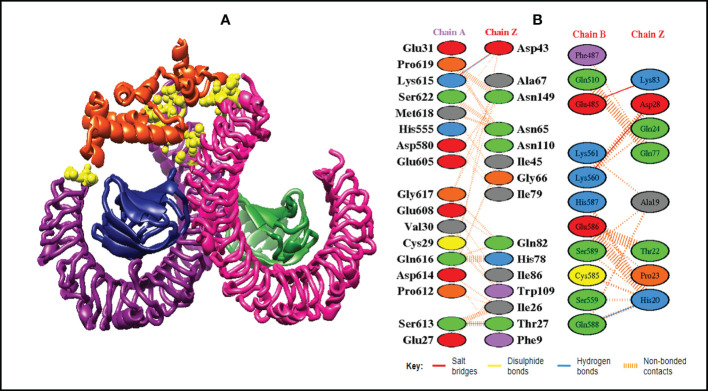
Schematic illustration of MEMPV-TLR4 docked cluster. **(A)** Docked pose of MEMPV-TLR4 docked cluster. Each chain of TLR4 receptor is represented in a different color, i.e. chain A: dark magenta, chain B: deep pink, chain C: navy blue, and chain D: forest green, construct in orange-red and interacting residues between immune receptor and vaccine are shown in yellow color. **(B)** Dimplot of vaccine construct and TLR4 docked complex.

In order to trigger the best immune reactions, a vaccine need to have a strong affinity for the host’s immunological receptors, such as Major Histocompatibility Complex (MHC) molecules and toll-like receptors. In this investigation, protein-protein blind molecular docking experiments were carried out using the PatchDock server (40) between the intended construct and the MHC I (PDB ID: 1I1Y), MHC II (PDB ID: 1KG0), TLR3 (PDB ID: 2A0Z) and TLR4 (PDB ID: 4G8A) molecules. The top ten docked clusters obtained *via* PatchDock were the subjected to FireDock refinement analysis (41). **Table 4** displays the docking outcome data for the top 10 complexes for all 4 immune cell receptors. We employed the PDBsum website (65) to obtain substantial knowledge about the residues that bind vaccine and receptor molecules. PDBsum’s characterization of prot-prot interactions indicates that the MEMPV interacting with MHC I receptor form 237 non-bonded contacts, 3 salt bridges, 6 hydrogen bonds, and no disulfide bonds (**Figure 5**). On the other hand, only one salt bridge, 72 non-bonded contacts, no disulfide bond and one hydrogen bond was found between MEMPV-MHC II interacting residues (**Figure 6**). Four hydrogen bonds, 83 non-bonded contacts, no disulfide bond and one salt bridge exist between binding atoms of MEMPV-TLR3 docked complex (**Figure 7**). TLR4 immune receptor contains 4 chains, however vaccine was found to be interacting with its two chains only i.e A and B. One salt bridge, two hydrogen bonds, 111 non-bonded contacts were observed between chain A (TLR4) and MEMPV, however, two salt bridges, one hydrogen bond and 65 non-bonded contacts were witnessed between Chain B (TLR4) and MEMPV. No disulfide bond was formed between interacting residues of MEMPV and TLR4 receptor (**Figure 8**).

**Table 4:** Molecular docking results of MEMPV with MHC I, MHC II, TLR3 and TLR4 molecules.

			MHC I			
Rank	Solution Number	Global Energy	Attractive VdW	Repulsive VdW	ACE	HB
1	5	-18.25	-31.73	13.50	8.92	-2.58
2	8	-0.66	-2.96	0.00	4.23	0
3	4	3.61	-11.35	3.42	6.11	-0.51
4	3	8.37	-37.09	8.76	11.57	-3.17
5	1	11.20	-52.43	45.96	23.47	-6.35
6	9	16.96	-4.12	4.50	7.01	0
7	7	51.02	-18.40	6.07	17.44	-4.27
8	2	89.34	-40.47	113.14	16.22	-3.27
9	6	228.18	-19.86	310.89	8.70	-2.56
10	10	1797.42	-57.63	2294.65	20.38	-3.41
**MHC II**						
**Rank**	**Solution Number**	**Global Energy**	**Attractive VdW**	**Repulsive VdW**	**ACE**	**HB**
1	9	-30.00	-36.40	18.65	2.29	-4.38
2	4	-15.52	-44.56	27.61	15.43	-4.52
3	10	-2.00	-18.61	11.01	8.11	-1.98
4	6	0.47	-28.62	13.98	11.85	-1.17
5	8	4.11	-25.22	22.20	11.05	-4.87
6	1	5.27	-10.62	1.57	7.71	-1.3
7	2	7.67	-31.51	30.26	8.42	-1.01
8	7	14.91	-15.17	11.48	11.03	-3.82
9	5	16.04	-1.21	0.00	2.11	-0.4
10	3	42.84	-44.22	69.16	17.39	-2.9
**TLR3**						
**Rank**	**Solution Number**	**Global Energy**	**Attractive VdW**	**Repulsive VdW**	**ACE**	**HB**
1	8	-23.73	-50.44	29.91	20.89	-4.81
2	5	-7.49	-24.30	8.36	8.59	-1.95
3	1	-1.51	-7.01	0.12	2.63	-1.85
4	6	12.09	-30.04	21.01	15.38	-3.05
5	10	14.23	-27.47	11.09	13.85	-1.78
6	7	17.27	-26.00	7.21	18.52	-3.7
7	3	24.07	-24.65	11.84	10.90	-2.51
8	9	28.09	-6.96	0.67	7.43	-1.6
9	2	426.23	-27.26	550.09	13.97	-6.66
10	4	4270.77	-51.42	5405.94	2.73	-6.72
**TLR4**						
**Rank**	**Solution Number**	**Global Energy**	**Attractive VdW**	**Repulsive VdW**	**ACE**	**HB**
1	9	-6.59	-29.61	9.50	8.83	-1.62
2	3	6.06	-1.08	0.00	0.40	0
3	5	7.10	-59.72	51.65	20.12	-5.25
4	10	7.34	-29.27	36.85	6.04	-5.67
5	7	8.20	-0.00	0.00	0.00	0
6	4	26.77	-28.04	9.18	15.78	-1.84
7	1	37.17	-62.43	172.07	1.57	-5.64
8	8	43.62	-17.28	26.71	9.73	-3.09
9	6	50.46	-41.14	139.91	9.06	-6.43
10	2	141.28	-23.45	200.18	6.74	-1.99

### Analysis of structural dynamics and stability of the docked complexes

**Figure 9:**
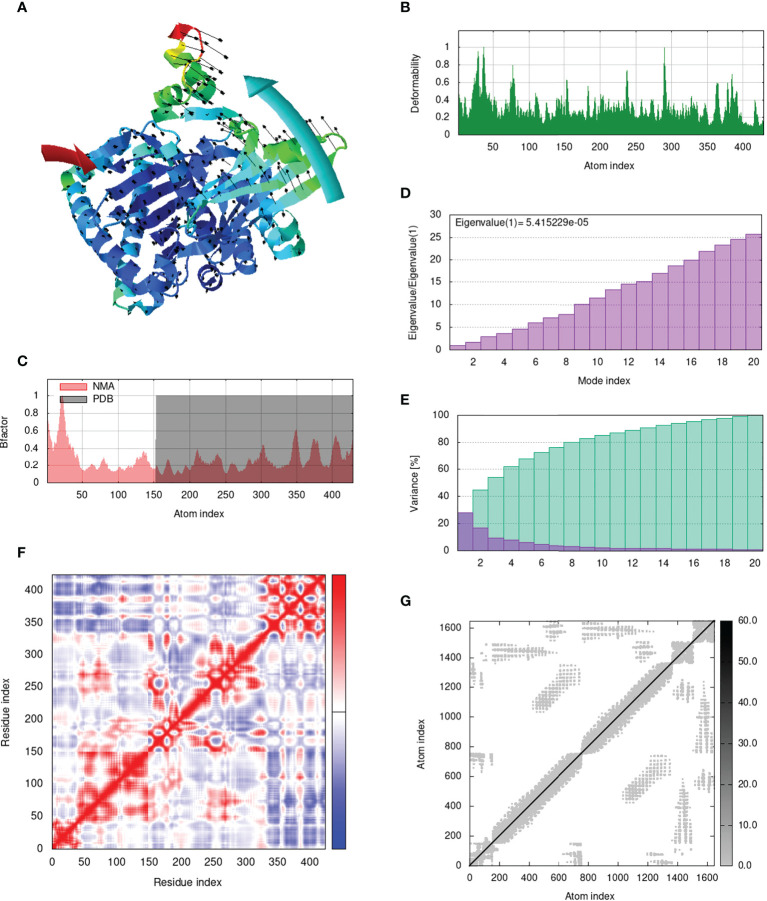
Analysis of the vaccine-MHC I complex in normal mode. **(A)** Structural strength of the refined protein-protein complex mobility; **(B)** deformability in relation to atoms; **(C)** B-factor in relation to atoms; **(D)** eigenvalue in relation to modes; **(E)** Structural variance; **(F)** covariance in relation to residue; **(G)** elastic network in relation to atoms.

**Figure 10:**
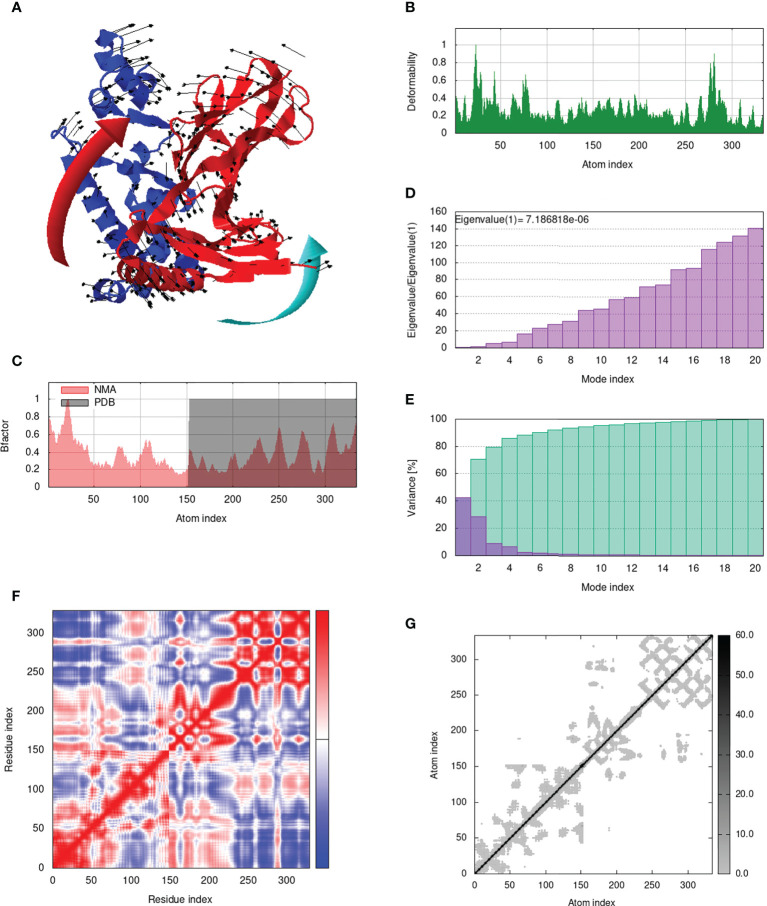
Analysis of the vaccine-MHC II complex in normal mode. **(A)** Structural strength of the refined protein-protein complex mobility; **(B)** deformability in relation to atoms; **(C)** B-factor in relation to atoms; **(D)** eigenvalue in relation to modes; **(E)** Structural variance; **(F)** covariance in relation to residue; **(G)** elastic network in relation to atoms.

**Figure 11:**
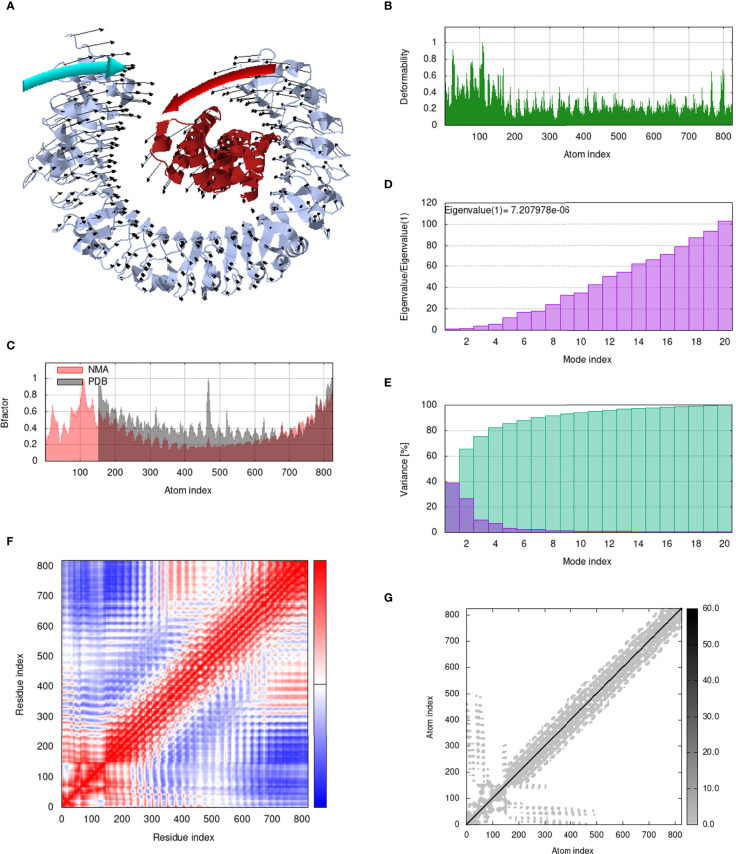
Analysis of the vaccine-TLR3 complex in normal mode. **(A)** Structural strength of the refined protein-protein complex mobility; **(B)** deformability in relation to atoms; **(C)** B-factor in relation to atoms; **(D)** eigenvalue in relation to modes; **(E)** Structural variance; **(F)** covariance in relation to residue; **(G)** elastic network in relation to atoms.

**Figure 12:**
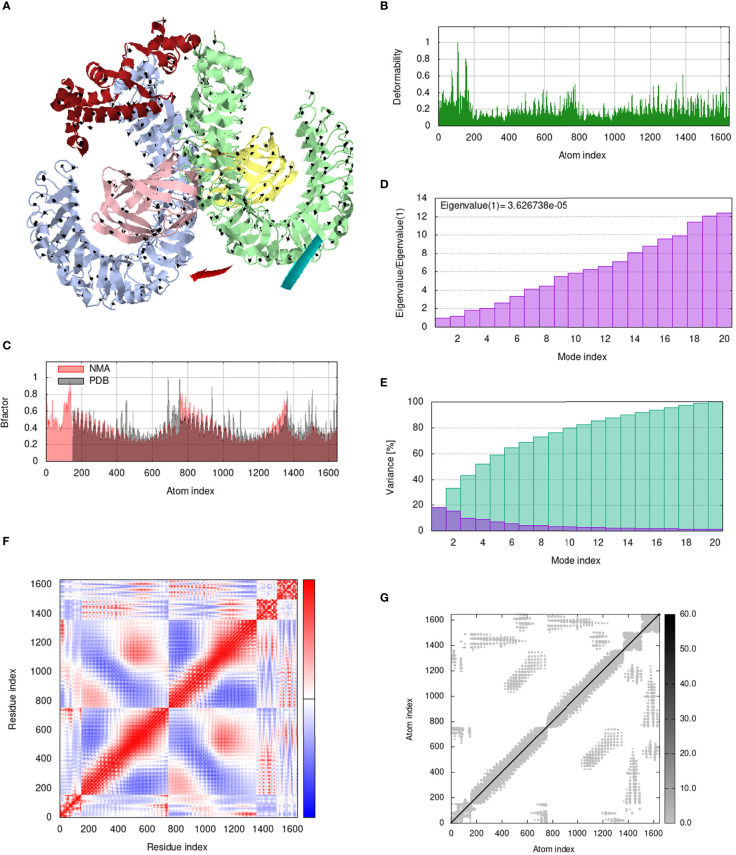
Analysis of the vaccine-TLR4 complex in normal mode. **(A)** Structural strength of the refined protein-protein complex mobility; **(B)** deformability in relation to atoms; **(C)** B-factor in relation to atoms; **(D)** eigenvalue in relation to modes; **(E)** Structural variance; **(F)** covariance in relation to residue; **(G)** elastic network in relation to atoms.

IModS adjusts the complex’s force field with regard to various time intervals in order to perform structural analysis of top hit docked complexes. The PDB complex internal coordinates were taken into account throughout this process. Arrows indicate the direction of the residue, and the length of the line depicts the degree of mobility in the three-dimensional model. It was discovered that both proteins (i.e. immune receptor and MEMPV construct) were mobile, allowing their chains to directly face one another **(** **Figures 9A**, **10A**, **11A**, **12A**. The produced docked models exhibit reduced distortion at each residue’s capacity level **(** **Figures 9B**, **10B**, **11B**, **12B**. B factor values align and endorse the results of RMSD **(** **Figures 9C**, **10C**, **11C** **12C**.The eigenvalue for MHC I-MEMPV docked cluster, MHC II-MEMPV docked cluster, TLR3-MEMPV docked cluster and TLR4-MEMPV docked cluster is 5.415229e−05, 7.186818e−06, 7.207978e−06 and 3.626738e−05 respectively **(** **Figures 9D**, **10D**, **11D**, **12D**. These decreased Eigen values and B factor values suggest that less energy is required to structurally adjust vaccine construct and immune receptor to bind with each other and generate immune response. Enhanced interactions between interfacing residues were demonstrated by low RMSD and highly correlated areas in all heat maps **(** **Figures 9** **-** **12** **)**. It is well known that the eigenvalue and the normal mode variance display an inverse relationship **(** **Figures 9E**, **10E**, **11E**, **12E**) (50, 66). Additionally, the covariance matrix provides a visual representation of coupling between residual pairs **(** **Figures 9F**, **10F**, **11F**, **12F** showing that this coupling may be due to correlated, uncorrelated, or anti-correlated motions. An elastic network model was produced using NMA at the end (**Figures 9G**, **10G**, **11G**, **12G**. The atom pairs connected by springs were discovered using this model. Each dot in the illustration represents a spring between the appropriate atomic pairs, and the amount of stiffness was taken into consideration while coloring. The darker the greys, the firmer the springs are.

### Estimation of codon adaptation index and *in silico* cloning

**Figure 13:**
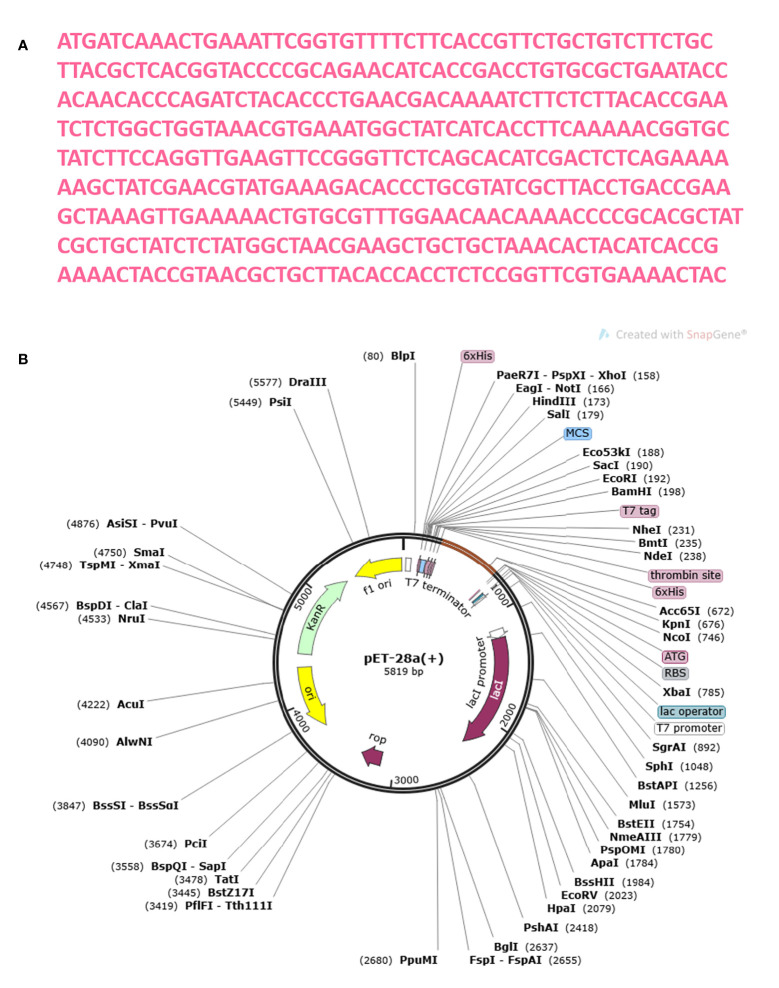
Optimization of codon and Computational cloning. **(A)** The reverse-translated DNA sequence of the MEMPV; **(B)** The MEMPV was cloned *in silico* into the pET28a expression vector.

The MEMPV construct sequence’s reverse translation was carried out using the JCat server in order to boost expression in *E. coli* (67). Using codon optimization, the recombinant vaccine protein was produced in the *E. coli* K12 system at a significantly greater level. **Figure 13A** shows the reverse translated and optimized sequence consisting of 450 nucleotides. The modified sequence’s codon adaptation index (CAI) value was determined to be 1.0 and its GC content to be 47.336. The fact that each of these figures fell within a reasonable range suggests that the MEMPV construct can be successfully expressed in the E. coli expression system. In order to confirm the JCat results, the sequence was then computationally cloned in the pET28a expression vector **(** **Figure 13B**).

### 3.11 Virtual immune simulation (IS) of MEMPV

**Figure 14:**
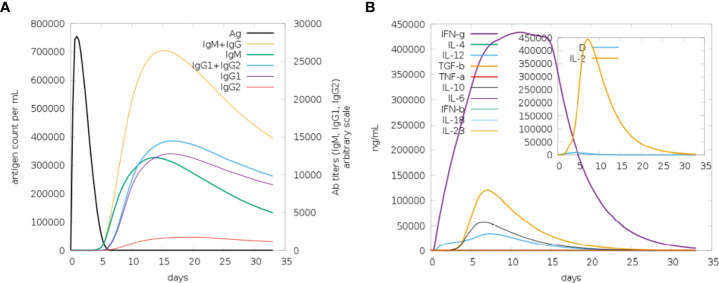
Findings of C-Immune Simulation. **(A)** Represent various types of immune responses generated against proposed vaccine **(B)** Represent prediction of interferons and interleukins induction in response to proposed vaccine.

Immune simulation was carried out using the C-IMMSIM server to anticipate how well the host immune system would respond to our developed MEMPV construct (54). All primary, secondary, and tertiary immune responses were produced in response to designed vaccine. According to **Figure 14A**, the combination of IgM and IgG antibodies were found in the highest amount, followed by IgG1+IgG2, IgG1, IgM and IgG2. Additionally, analysis and prediction of interleukin and cytokine induction were performed **(** **Figure 14B** **)**. All of these findings demonstrate immunogenic and antigenic nature of our developed MEMPV construct.

## Discussion

Multi-epitope vaccines act as a potential yet viable solution to combat emerging infectious diseases due to their capacity to elicit cell mediated and humoral immune response simultaneously. The newly discovered monkeypox virus is a zoonotic orthopoxvirus that infects *homo sapiens* and culminates in illnesses resembling smallpox. Uptil now, no promising therapeutic is available to treat MPXV infections aggravating the need to establish a next generation, state of the art, multi-valent peptide based vaccine. Multi-valent vaccines can elicit specific immune responses based on conserved epitopes in entire antigenic sequences, avoiding reactions against un-favourable epitopes that might result in immunological-pathogenic or immune-modulating reactions against the host (68, 69). A plausible candidate for vaccine development is the cell surface binding protein, which is expressed in the outer membrane of the microbe and facilitates virion attachment to the target cell by attaching to chondroitin sulfate on the cell surface. The current study establishes the concept of a multi-epitope vaccine formed from a single protein using biophysical and bioinformatics approach.

FASTA Sequence of Cell surface binding protein retrieved from Uniprot protein database was subjected to immunogenic profiling. Physicochemical analysis and antigenic evaluation revealed the protein to be strongly immunogenic, non-allergic, virulent, non-toxic and stable enough for *in vitro* experimentation and epitope mapping. B cell derived T cell epitopes were mapped against the targeted protein and subjected to iterative and subtractive proteomics pipeline. Only those epitopes were chosen for MEMPV assembly that exhibited substantial affinity for the DRB1*0101 allele in competitive binding assays with an IC50 value less than 100nM, as well as being non-allergic, highly antigenic, virulent, non-toxic, IFN-gamma positive and water soluble. Screened epitopes were joined together *via* adjuvant and linkers to enhance the efficacy of the finalized MEMPV construct. In order to ensure the immunogenic nature of designed construct, physicochemical profiling, antigenicity anticipation and prediction of allergenicity was performed again. Designed MEMPV was found to be stable, strongly antigenic and immunogenic making it a potential therapeutic to combat against monkeypox infection.

The study of interactions between antigens and receptor immune molecules is crucial for the formulation of vaccines. The vaccine’s 3D architecture was anticipated and then further enhanced *via* subsequent refinement. The rigorous 3D structural analysis demonstrated the proposed vaccine prototype’s structural stability and revealed that highest proportion of residues lie in the favourable region of Ramachandran plot. Furthermore, according to the anticipated instability score, the developed vaccine structure will be perfectly robust when expressed, increasing its potential as a vaccine. One of the most important steps in validating a nascent vaccine (70), which must be translated in an appropriate expression system, is the confirmation of immune-reactivity based on serological analysis. The creation of recombinant peptides is thought to be best accomplished using the E. coli expression system (71, 72). The hypothesized construct interacted strongly with immune receptors like MHC I, MHC II, TLR3 and TLR4 in molecular docking assay, demonstrating the immunogenic nature of the suggested construct. MD simulations were used to verify the vaccine docked complex’s stability. The molecular stability of the multi-epitope vaccine complex in a cellular context was ensured by this investigation, which supported the vaccine’s strong molecular interactions with the immune receptor. This suggests that the vaccine construct developed in this study has the ability to induce robust immune responses with high gene expression.

Theoretically, considerable cellular and humoral immune responses ought to be elicited by the MEMPV since it was created by combining several B derived T cell epitopes. However, depending on a variety of elements, including the pathogen’s mechanism, the immune system response may change (73). Consequently, host immune simulation response analysis was performed on the vaccine formulation (74). All immunological responses—primary, secondary, and tertiary—were triggered by the intended vaccine. The largest concentration of IgM+IgG antibodies was discovered, followed by IgG1+IgG2, IgG1, IgM, and IgG2. Interleukin and cytokine induction analysis and prediction were also carried out. These results demonstrate that our proposed MEMPV construct is strongly immunogenic, however, this prophylactic vaccination need to be tested experimentally against monkeypox virus to gauge its effectiveness and safety.

## Conclusion

Monkeypox viral disease is an emerging global threat characterized by fever, rash, headache, flu and lymphadenopathy. In order to provide a promising solution to tackle the disease, cell surface binding protein of monkeypox virus was employed to design state of the art, next generation, multi-antigenic vaccine construct *via* immune-informatics and biophysical approaches. Assessment of physicochemical properties, structural flexibility, antigenicity, allergenicity, virulence, toxicity and solubility validated the immunogenicity of the hypothesized vaccine construct. Molecular docking studies, molecular dynamic simulations and c-immune simulations revealed that the designed vaccine has the potential to strongly elicit cell mediated and humoral immune response. The proposed model is ready to be employed by experimental vaccinologists for additional *in vitro* and *in vivo* tests to validate its response against monkeypox disease.

## Data availability statement

The datasets presented in this study can be found in online repositories. The names of the repository/repositories and accession number(s) can be found in the article/**Supplementary Material.**

## Author contributions

MY: Designed research framework, performed all assays and tests and composed manuscript. SI: Composition and refinement of the manuscript AU: Manuscript refinement and editing SB: Writing, reviewing and editing of the manuscript. All authors contributed to the article and approved the submitted version.

## Conflict of interest

The authors declare that the research was conducted in the absence of any commercial or financial relationships that could be construed as a potential conflict of interest.

## Publisher’s note

All claims expressed in this article are solely those of the authors and do not necessarily represent those of their affiliated organizations, or those of the publisher, the editors and the reviewers. Any product that may be evaluated in this article, or claim that may be made by its manufacturer, is not guaranteed or endorsed by the publisher.
