# Supplementary material for: Immuno-informatics profiling of monkeypox virus cell surface binding protein for designing a next generation multi-valent peptide-based vaccine
Source: Front Immunol. 2022 Nov 2;13:1035924. doi: 10.3389/fimmu.2022.1035924 (PMC9668073; doi:10.3389/fimmu.2022.1035924)
Supplement: Supplementary file 1 [file DataSheet_1.docx]

**Immuno-informatics Profiling of Monkeypox Virus Cell Surface Binding Protein for Designing a Next Generation Multi-Valent Peptide-based Vaccine.**

**Maha Yousaf^1,*^, Saba Ismail^2^, Asad Ullah^3^, Shabana Bibi^4,5,*^**

1. Department of Biosciences, COMSATS University Islamabad, Islamabad 45550, Pakistan; [maha.yousaf.vt8086@iiu.edu.pk](mailto:maha.yousaf.vt8086@iiu.edu.pk)
2. Department of Biological Sciences, National University of Medical Sciences, Rawalpindi 46000, Pakistan; [sabaismail7@gmail.com](mailto:sabaismail7@gmail.com)
3. Department of Health and Biological Sciences, Abasyn University, Peshawar 25000, Pakistan; [asad.ullah@abasyn.edu.pk](mailto:asad.ullah@abasyn.edu.pk)
4. Department of Biosciences, Shifa Tameer-e-Millat University, Islamabad 44000, Pakistan; [shabana.bibi.stmu@gmail.com](mailto:shabana.bibi.stmu@gmail.com)
5. Yunnan Herbal Laboratory, College of Ecology and Environmental Sciences, Yunnan University,

Kunming 650091, China; [shabana_bibi@ynu.edu.cn](mailto:shabana_bibi@ynu.edu.cn)

*** Corresponding Author:**

**Maha Yousaf**

Department of Biosciences

COMSATS University Islamabad

Pakistan

[maha.yousaf.vt8086@iiu.edu.pk](mailto:maha.yousaf.vt8086@iiu.edu.pk)

**Dr. Shabana Bibi**

Department of Biosciences

Shifa Tameer-e-Millat University, Islamabad

Pakistan

[shabana.bibi.stmu@gmail.com](mailto:shabana.bibi.stmu@gmail.com)

**Supplimentary File**

**Table 1:** Description anticipated B cell peptides.

| **Serial** | **start** | **end** | **peptide** | **Length** |
| --- | --- | --- | --- | --- |
| 1 | 5 | 7 | LSP | 3 |
| 2 | 26 | 33 | IHYNESKP | 8 |
| 3 | 35 | 37 | TIQ | 3 |
| 4 | 73 | 86 | EDDYGSNHLIDVYK | 14 |
| 5 | 98 | 110 | KKKYSSYEEAKKH | 13 |
| 6 | 129 | 129 | V | 1 |
| 7 | 140 | 151 | SIRSANMSAPFD | 12 |
| 8 | 155 | 164 | YLDNLLPSTL | 10 |
| 9 | 171 | 178 | GTTINHSA | 8 |
| 10 | 203 | 227 | LSSSNHEGKPHYITENYRNPYKLND | 25 |
| 11 | 234 | 250 | SGEIIRAATTSPVRENY | 17 |
| 12 | 268 | 271 | KYIE | 4 |
| 13 | 300 | 300 | R | 1 |

**Table 2:** Description anticipated B cell based T cell epitopes

| **B Cell Based T cell Epitopes** | **Percentile rank** | **MHC Pred (IC 50)** | **Antigenicity** | **Allergenicity** | **Virulence** | **Toxin-pred** | **Water Solubility** | **ifn vs other cytokines** | **Selection** |
| --- | --- | --- | --- | --- | --- | --- | --- | --- | --- |
| EDDYGSNHL | 2.1 | 49.09 | 0.3606 | - | - |  |  |  |  |
| SNHLIDVYK | 1.5 | 67.3 | -0.1161 | - | - |  |  |  |  |
| KKKYSSYEE | 8 | 339.63 | - | - | - |  |  |  |  |
| SSYEEAKKH | 0.84 | 17.3 | 0.1447 | - | - |  |  |  |  |
| SIRSANMSA | 0.12 | 48.31 | 1.5362 | allergen | - |  |  |  |  |
| SANMSAPFD | 15 | 27.61 | 0.3804 | - | - |  |  |  |  |
| PHYITENYR | 3.6 | 46.88 | 0.7327 | allergen | - |  |  |  |  |
| NYRNPYKLN | 9.7 | 169.43 | - | - | - |  |  |  |  |
| LSSSNHEGK | 0.9 | 926.83 | - | - | - |  |  |  |  |
| EGKPHYITE | 2 | 1517.05 | - | - | - |  |  |  |  |
| HYITENYRN | 6.4 | 46.13 | 0.5818 | non-allergen | Virulent 1.0606 | Non-toxin | Good water solubility | positive 0.42164733 | Selected |
| YRNPYKLND | 40 | 4.7 | 0.4510 | allergen | - |  |  |  |  |
| SGEIIRAAT | 21 | 162.93 | - | - | - |  |  |  |  |
| AATTSPVRE | 17 | 37.67 | 0.3180 | - | - |  |  |  |  |
| GEIIRAATT | 1.7 | 348.34 | - | - | - |  |  |  |  |
| ATTSPVREN | 7.6 | 190.11 | - | - | - |  |  |  |  |
| EIIRAATTS | 2.7 | 8.04 | 0.0206 | - | - |  |  |  |  |
| TTSPVRENY | 0.05 | 52.48 | 0.7917 | non-allergen | Virulent 1.0575 | Non-toxin | Good water solubility | positive 0.64719524 | Selected |

**Reference Set of MHC I Alleles:**

A set of reference alleles to which these chosen epitopes interact with are: HLA-A*01:01, HLA-A*02:01, HLA-A*02:01, HLA-A*02:03, HLA-A*02:03, HLA-A*02:06, HLA-A*02:06, HLA-A*03:01, HLA-A*03:01, HLA-A*11:01, HLA-A*11:01, HLA-A*23:01, HLA-B*08:01, HLA-A*23:01, HLA-A*24:02, HLA-A*24:02, HLA-A*26:01, HLA-A*26:01, HLA-A*30:01, HLA-A*30:01, HLA-B*57:01, HLA-A*30:02, HLA-A*31:01, HLA-B*58:01, HLA-A*32:01, HLA-A*33:01, HLA-A*33:01, HLA-A*68:01, HLA-A*68:01, HLA-A*68:02, HLA-A*68:02, HLA-A*30:02, HLA-B*07:02, HLA-B*51:01, HLA-B*07:02, HLA-B*08:01, HLA-B*15:01, HLA-B*15:01, HLA-B*35:01, HLA-A*31:01, HLA-B*35:01, HLA-B*40:01, HLA-B*40:01, HLA-B*44:02, HLA-B*44:02, HLA-B*44:03, HLA-B*44:03, HLA-B*51:01, HLA-A*01:01, HLA-B*53:01, HLA-B*53:01, HLA-B*57:01, HLA-A*32:01, and HLA-B*58:01.

**Reference Set of MHC II Alleles:**

HLA-DRB4*01:01, HLA-DRB1*04:01, HLA-DRB1*04:05, HLA-DRB1*07:01, HLA-DRB1*09:01, HLA-DRB1*11:01, HLADRB1*03:01, HLA-DRB1*13:02, HLA-DRB1*15:01, HLA-DRB3*01:01, HLA-DRB1*12:01, HLA-DRB3*02:02, HLA-DRB1*08:02, HLA-DRB1*01:01, and HLA-DRB5*01:01.
